# Genomics and Metabolomics in Obesity and Type 2 Diabetes

**DOI:** 10.1155/2016/9415645

**Published:** 2016-05-25

**Authors:** Adam Kretowski, Francisco J. Ruperez, Michal Ciborowski

**Affiliations:** ^1^Department of Endocrinology, Diabetology and Internal Medicine, Medical University of Bialystok, Sklodowskiej-Curie 24a, 15-276 Bialystok, Poland; ^2^Clinical Research Centre, Medical University of Bialystok, Sklodowskiej-Curie 24a, 15-276 Bialystok, Poland; ^3^Center for Metabolomics and Bioanalysis (CEMBIO), Faculty of Pharmacy, University San Pablo-CEU, Montepríncipe Campus, Boadilla del Monte, 28668 Madrid, Spain

Obesity and associated diseases are responsible for the global burden of morbidity and mortality, consequently having a huge impact on the economy of healthcare system. Obesity is the leading risk factor for type 2 diabetes (T2DM), and both conditions currently affect millions of individuals worldwide, being considered as epidemic. The risk for development of obesity and consequently T2DM depends on multiple factors, such as genetic susceptibility [1], composition of the gut microbiota [2], or environmental factors, that is, increased consumption of “unhealthy” food, rich in either saturated fat or refined carbohydrates, as well as reduced physical activity or exposition to pesticides, phenols, phthalates, or heavy metals [3, 4]. Over the past decade a number of common genetic susceptibility loci for obesity and T2DM have been identified [5, 6] and the estimated heritability of obesity/T2DM ranges from 20% to 80% [7, 8]. However, considering the rapid rise of obesity prevalence in the last decade and the radical differences in BMI among individuals living in the same “obesity promoting” environment, it can be suggested that the risk for obesity development depends on complex interactions between genes and the environment [4].

The metabolome is influenced by genetic variants, epigenetic factors, changes in gene expression, or enzyme activity, as well as by environmental factors (diet, physical activity, and pharmaceuticals) and aging [9]. Consequently, metabolomics is a valuable tool to study environment-related modifications of the genetic susceptibility. Currently, based on metabolomics studies, several small molecules are proposed as related to diabetes and/or obesity [10], and the utility of metabolomics for diet-related studies has already been shown [11]. However, as the prevalence of obesity is continually increasing, more research is needed for better understanding of the mechanisms of obesity and the evolution to T2DM. Moreover, novel treatment strategies for obesity/T2DM are sought, while the already known effective treatments such as life style modification (physical activity, diet) or weight loss surgeries should be studied in more detail to explore the molecular pathways lying underneath the weight loss and the T2DM remission.

This special issue is devoted to genetic and molecular biomarkers of obesity and diabetes, as well as to studies in which novel holistic approaches are applied to improve the knowledge on molecular aspects of the evolution or treatment of obesity and diabetes.

Among small molecule metabolites, branched-chain amino acids and acylcarnitines have been linked to obesity-associated insulin resistance (IR). C. Hellmuth et al. performed a longitudinal study on obese children participating in a lifestyle intervention trial for inducing weight loss, to explore changes in amino acids, carnitines, and insulin resistance after the intervention. Only tyrosine was significantly associated with HOMA before and after the intervention and was found to change during the intervention. In addition, BCAA levels were negatively related to insulin resistance in cross-sectional analyses but not in the longitudinal profiling. The authors concluded that tyrosine alterations in association with insulin resistance precede the alteration in BCAA metabolism.

Another group of metabolites shown to be altered in diabetes are short-chain fatty acids (SCFAs). In their article, E. Lachmandas et al. showed the relationship between diabetes, SCFAs, and increased susceptibility to tuberculosis. Diabetes is associated with a threefold increased risk of tuberculosis, while butyrate producing bacteria are known to be decreased in gut microbiota of patients with diabetes. The authors linked these observations and performed several experiments in which they showed that SCFAs exhibit anti-inflammatory properties, while low doses of butyrate decreased* Mycobacterium tuberculosis*-induced proinflammatory cytokine responses and increased production of IL-10. IL-10 may play a role in mediating the inhibitory effects of butyrate on the host immune response to* M. tuberculosis.*

M.-O. Guzmán-Ornelas et al. investigated the relationship between CCL2 G-2518A and CCR2Val64Ile polymorphisms and the levels of soluble chemokine (C-C motif) ligand-2 (sCCL2), metabolic markers, and adiposity in a Mexican population with insulin resistance. The CCL2 polymorphism was found to be associated with IR, and the CCL2-phenotype carriers (A+) had lower body mass and fat indexes, insulin, and HOMA-IR, as well as higher adiponectin levels. Furthermore, individuals with IR presented higher sCCL2 level. The double-polymorphic phenotype carriers (A+/Ile+) exhibited higher sCCL2 than double wild type-phenotype carriers (A−/Ile−). The present findings allow the authors to suggest a possible association of sCCL2 production with the adiposity and polymorphic phenotypes of CCL2 and CCR2, in Mexican-Mestizos with IR.

Other articles included in this special issue discuss the treatment of obesity and diabetes. Although several natural (green tea polyphenols, cocoa, and chitin/chitosan) antiobesity agents have been already proposed, researchers are still interested in finding phytochemical strategies to treat obesity. N. G. S. Jambocus et al. used an NMR-based metabolomics approach to evaluate mechanisms of antiobesity effect of one extract from the leaves of* Morinda citrifolia *L. The study was performed on an animal model of obesity, Sprague-Dawley rats with high fat diet. Changes in metabolic pathways such as glucose metabolism and TCA cycle, amino acid, choline, creatinine, and gut microbiome metabolism were observed. Treatment with* M. citrifolia *L. leaves extract resulted in significant improvement in the metabolic perturbations caused by obesity.

However, in the case of morbidly obese individuals, more radical interventions are necessary to overcome diabetes. Currently, the most effective treatment for obesity and type 2 diabetes is bariatric surgery. In the article by K. Sarosiek et al., nontargeted global metabolomics analysis was performed to evaluate changes occurring in nondiabetic and type 2 diabetic patients experiencing standard preoperative liquid weight loss diet, less extreme sleeve gastrectomy, or a full gastric bypass surgery. Preoperative weight loss diet was associated with strong changes in lipid metabolism showing glucose usage that shifted away from glycolytic pyruvate production towards pentose phosphate pathway, via glucose-6-phosphate. These modifications appeared to be shared by all patients regardless of T2D status or bariatric surgery procedure. Results obtained by the authors suggest that bariatric surgery might promote antioxidant defense and insulin sensitivity through increased heme synthesis and HO activity or expression. Other changes in histidine and its metabolites following surgery might indicate alteration in gut microbiome ecology or liver function.

Articles included in this special issue cover a broad range of topics, which we hope the readers of the journal will find important and interesting.

*Adam Kretowski* *Adam Kretowski* *Francisco J. Ruperez* *Francisco J. Ruperez* *Michal Ciborowski* *Michal Ciborowski*
